# Effect of the Design and Exposure Mode of Different Multiple-Peak Light-Curing Units on Light Emission, Temperature Rise, and Intraoral Access

**DOI:** 10.3290/j.jad.c_2598

**Published:** 2026-03-24

**Authors:** Maria Tereza Hordones Ribeiro, Guilherme Mendonça Benoni, Maribí Isomar Terán Lozada, Airin Karelys Avendaño Rondon, Gisele Rodrigues da Silva, Carlos José Soares

**Affiliations:** a Maria Tereza Hordones Ribeiro Dentist, Department of Operative Dentistry and Dental Materials, Dental School, Federal University of Uberlândia (UFU), Uberlândia, Minas Gerais, Brazil. Av. Pará, 1720, Bloco 4LA sala 4LA-42, Campus Umuarama, Uberlândia, Minas Gerais, Brazil. Experimental design, performed the experiment, wrote the manuscript.; b Guilherme Mendonça Benoni Dentist, Department of Operative Dentistry and Dental Materials, Dental School, Federal University of Uberlândia (UFU), Uberlândia, Minas Gerais, Brazil. Av. Pará, 1720, Bloco 4LA sala 4LA-42, Campus Umuarama, Uberlândia, Minas Gerais, Brazil. Performed the experiments and proofread the manuscript.; c Maribí Isomar Terán Lozada Dentist, Department of Operative Dentistry and Dental Materials, Dental School, Federal University of Uberlândia (UFU), Uberlândia, Minas Gerais, Brazil. Av. Pará, 1720, Bloco 4LA sala 4LA-42, Campus Umuarama, Uberlândia, Minas Gerais, Brazil. Consulted and performed statistical evaluation and contributed substantially to the discussion.; d Airin Karelys Avendaño Rondon Dentist, Department of Operative Dentistry and Dental Materials, Dental School, Federal University of Uberlândia (UFU), Uberlândia, Minas Gerais, Brazil. Av. Pará, 1720, Bloco 4LA sala 4LA-42, Campus Umuarama, Uberlândia, Minas Gerais, Brazil. Performed the experiments and proofread the manuscript.; e Gisele Rodrigues da Silva Dentist, Department of Operative Dentistry and Dental Materials, Dental School, Federal University of Uberlândia (UFU), Uberlândia, Minas Gerais, Brazil. Av. Pará, 1720, Bloco 4LA sala 4LA-42, Campus Umuarama, Uberlândia, Minas Gerais, Brazil. Idea, Experimental design, wrote the manuscript, proofread the manuscript and contributed substantially to the discussion.; f Carlos José Soares Dentist, Department of Operative Dentistry and Dental Materials, Dental School, Federal University of Uberlândia (UFU), Uberlândia, Minas Gerais, Brazil. Av. Pará, 1720, Bloco 4LA sala 4LA-42, Campus Umuarama, Uberlândia, Minas Gerais, Brazil. Idea, Experimental design, wrote the manuscript, proofread the manuscript and contributed substantially to discussion.

**Keywords:** beam profile, irradiance, light-curing unit, multiple peak, radiant exposuree

## Abstract

**Purpose:**

To measure the tip diameter (mm) and the ability to cover the anterior and posterior large restorations, radiant power (mW), radiant exitance (mW/cm²), emission spectrum (mW/cm²/nm), radiant exposure (J/cm²), the effect of the design on the access to the mouth posterior region, the temperature rise inside the pulp of three light-curing units (LCUs) and a new LCU available in the Brazil.

**Methods and Materials:**

Four LCUs that cost over US$900, three well-established (Bluephase G2, Ivoclar Vivadent; VALO Grand, Ultradent; and VALO Cordless, Ultradent), and a new LCU (Quazar, FGM) were tested in standard mode (20 s for all LCUs), high mode (3 s for VALO Cordless, 5 s for Quazar, and 20 s for Bluephase G2), and Xtra power mode (3 s for VALO Grand). The radiant power (mW) and emission spectrum (mW/nm) were measured using an integrating sphere connected to a fiberoptic spectroradiometer. The internal tip diameter (mm) of each LCU was measured using a digital caliper and was used to calculate the radiant exitance (mW/cm²). Radiant exitance profiles at the light tip were measured using a laser beam profiler. The radiant exposure (J/cm²) was calculated. The *in vitro* temperature rise produced by LCUs inside the pulp cavity of molar teeth was measured using a thermocouple. The mouth access of the LCU tip on the occlusal surface of the first mandibular molar tooth with two mouth openings of 25 mm and 45 mm at the incisors was evaluated. The cost of each LCU in Brazil was correlated with internal tip diameter, radiant power, and radiant exitance.

**Results:**

All the LCUs were multiple-peak LCUs, and a uniform output. Quazar, VALO Cordless, and VALO Grand could maintain a perpendicular position regardless of mouth interincisal opening, while the Bluephase G2 required a tip angulation of 31.6 degrees at the 25 mm interincisal opening. The VALO Grand and VALO Cordless produced the highest temperature rise in standard mode (≈2.5°C), while in high mode, all LCUs produced lower temperature increases that use 5 s for Quazar and 3 s for VALO Grand and VALO Cordless, except for Bluephase G2, which produced a higher temperature rise (≈2.0°C) when activated for 20 s. There was a positive correlation between the cost of these LCUs and their averaged radiant power, diameter and the radiant exitance.

**Conclusions:**

The LCUs tested emit light in the blue and violet spectra, characterizing them as multiple peaks. The temperature increases in the produced pulp remained within safe thermal limits (< 2.5°C), although standard-mode exposures produced higher pulp temperature rises. Bluephase G2 created higher angulation at 25 mm of interincisal mouth opening. The Quazar LCU produced a light output that was comparable to that from leading LCUs.

Resin-based restorative composites (RBCs) are widely used in modern dentistry.^9^ They offer versatile and practical solutions in direct and indirect restorative procedures.^9,39^ Light-curing units (LCUs) photo-cure these materials and are an essential part of the restorative process.^12,21,22,34^ These LCUs should deliver energy and wavelengths that are compatible with the manufacturer’s recommendations to ensure adequate mechanical properties and longevity of the restorations.^7,31^ Other important factors to consider include the power output, the emission spectrum, beam collimation, the ergonomics of the LCU, and whether it is easy to disinfect.^26,34,38^

First and second generation LED LCUs had a narrow emission spectrum with single peak emission in the blue wavelength range, corresponding to the absorption peak of camphorquinone (CQ).^5,6,19,40^ CQ is yellowish and can compromise the color of the restoration.^8,11,30,33^ The esthetic demands of whitened teeth and the popularization of bulk-fill RBCs stimulated the introduction of alternative photoinitiators, such as TPO (diphenyl(2,4,6-trimethylbenzoyl)phosphine oxide) and Ivocerin, which are used to overcome the yellow color of CQ and help increase the depth of cure.^16,17^ Not all RBCs require violet light for proper photo-curing; however, multiple peaks have gained in popularity for photo-curing a broader variety of materials,^3^ as it is propagated that they can better activate alternative photoinitiators in addition to CQ.^8,12^ The radiant spectrum of the different multiple-peak LCUs is only expressed as covering the blue and violet range; however, it is not always the case that the wavelength peak emission is the same for different LCUs.

Establishing a photoactivation protocol is essential for the success of the restoration and achieving adequate material polymerization.^21,34^ The LCU should be held perpendicular to the restorative material, with its tip as close as possible to the area to be photo-cured, ensuring this position throughout the LCU.^10^ This protocol can be compromised if the patient has a limited mouth opening, in posterior regions with poor visibility of the teeth, if the LCU has an angled or unergonomic body, or if the operator neglects any of these steps.^9,16,20,22,28^ These factors, alone or in combination, can reduce the radiant exitance by creating angles between the tip and the restoration, such as in the proximal boxes of Class II cavities, thereby compromising the longevity of the restoration.^16,20,40^

The diameter of the curing tip, radiant power, and spectral distribution directly influence the polymerization efficiency of RBCs. Depending on the manufacturer, commercial LCUs have different ergonomics, design, wavelength, energy, radiant exitance, and cost.^1,28^ Characterizing LCUs is essential as it allows individual performance to be assessed and helps the clinician choose the LCU that best suits their practice. Basic LCU characterization can be obtained using a dental radiometer or in a laboratory environment using a fiberoptic spectrometer attached to an integrating sphere.^25^ Although the fiberoptic spectrometer attached to an integrating sphere is expensive, it provides more accurate power values (mW) than a dental radiometer that also cannot provide the emission spectra (mW/cm²/nm).^39^ Only by obtaining this information and measuring the diameter of the active tip of the LCU is it possible to calculate radiant exitance (mW/cm²). The spectral radiant power (mW/nm) and radiant exposure (J/cm²) from the LCU.^9^ Additionally, accessibility to posterior regions in the mouth and the potential for pulp temperature rise are critical for clinical safety and performance. Despite the availability of various LCUs in the Brazilian market, there is limited evidence correlating their physical and optical properties with their cost.^29,36^ The Quazar LCU, manufactured by Guilin Woodpecker Medical Instrument, is sold by the FGM company, which is one of the leading companies for restorative and bleaching products in Brazil. The limited information about this LCU has raised questions about its performance compared with leading LCUs. This study aimed to characterize Quazar LCU compared with three LCUs available in Brazil by assessing their tip diameter (mm), radiant power (mW), radiant exitance (mW/cm²), emission spectrum (mW/cm²/nm), radiant exposure (J/cm²), the effect of their design on the access to the posterior teeth, their beam profile, pulp temperature rise, and correlate these characteristics with their market cost. The null hypotheses are:

1. There will be no difference in the tip diameter (mm), radiant power (mW), radiant exitance (mW/cm²), emission spectrum (mW/cm²/nm), the effect of the design on the access to the posterior region, beam profile, and pulp temperature rise of these four LCUs;
2. The market cost of these four LCUs will not influence the characteristics measured.

## MATERIALS AND METHODS

### Characterization of the LCUs

**Fig 1:**
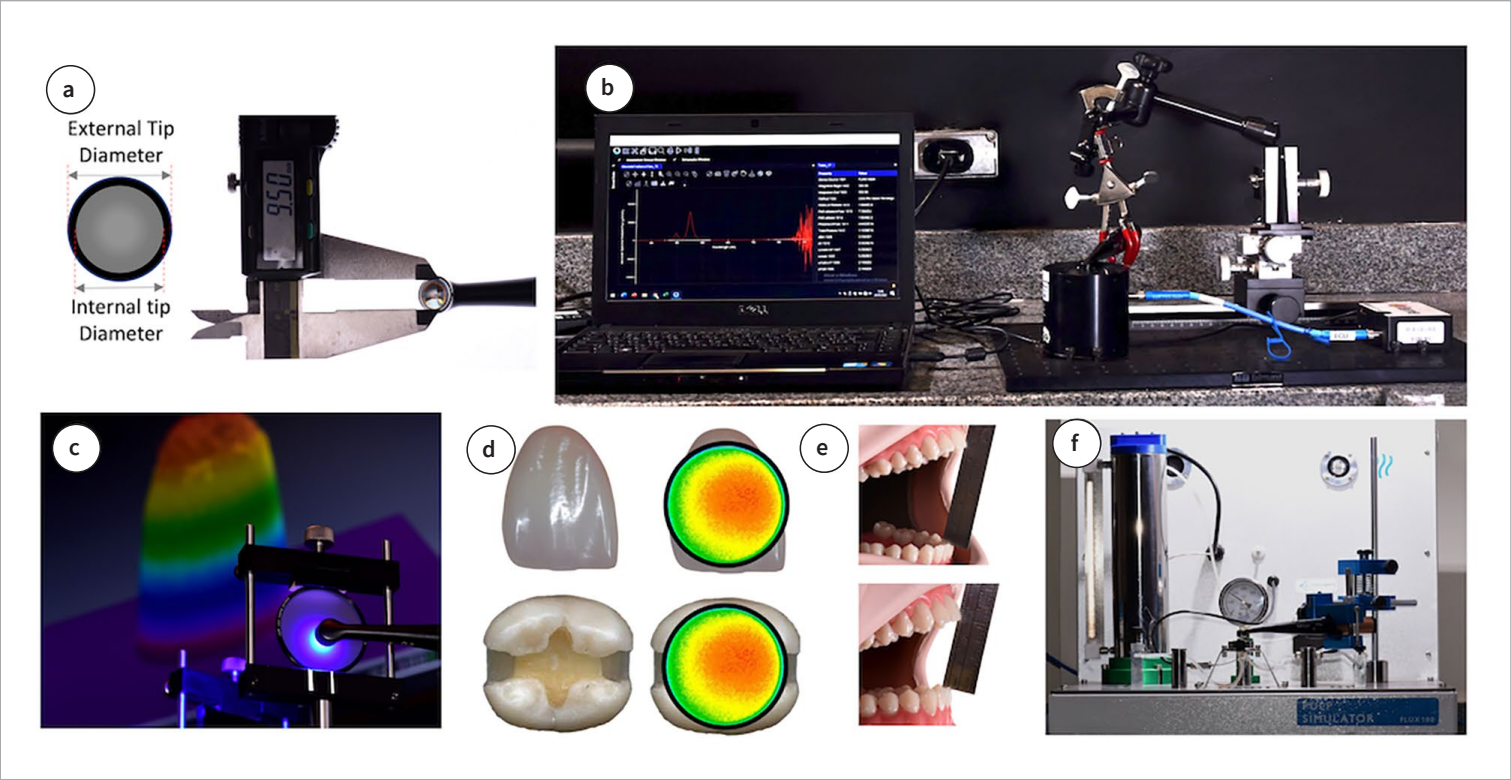
Methodologies used in this study: (a) measurement of the LCU tip diameter; (b) integrating sphere; (c) beam profile; (d) overlapping the LCU tip diameter with anterior and posterior teeth; (e) mouth opening setup; (f) temperature machine Flux100.

Four LCUs purchased in Brazil were tested: Quazar (FGM, Joinville, SC, Brazil); Bluephase G2 (Ivoclar Vivadent, Schaan, Liechtenstein), VALO Cordless (Ultradent, South Jordan, USA), and VALO Grand (Ultradent) (Table 1). The LCUs were evaluated in two light-curing modes: standard and high for Quazar, Bluephase G2, and VALO Cordless; standard and Xtra for VALO Grand. The LCU’s external and internal tip diameters were measured using a digital caliper (Mitutoyo, Tokyo, Japan) (Fig 1a). The tip area was calculated from the internal diameter of the LCU tip.^14^

**Table 1:** Light-curing units information

LCU	Serial number	LCU type – wavelength emission	External tip diameter (mm)	Internal tip diameter (mm)	Cost (US$)	Battery/mains	Tip-light conductor	Manufacturer
Quazar	L2320259L	LED – multi-peak	13.0	9.9	1139	Battery	None	FGM, Joinville, SC, Brazil
Bluephase G2	1404000004	LED – multi-peak	9.8	9.0	876	Battery	Optical fiber with mixer – black	Ivoclar Vivadent, Schaan, Liechtenstein
VALO Cordless	C43122	LED – multi-peak	13.1	9.7	1394	Battery	None	Ultradent, South Jordan, UT, USA
VALO Grand	MFG3227-5	LED – multi-peak	15.1	11.8	1515	Battery	None	Ultradent, South Jordan, UT, USA


### Emission Spectrum and Power

The emission spectrum (mW/nm) and the radiant power (mW) emitted from each LCU were measured five times using an integrating sphere (Labsphere, North Sutton, NH, USA) attached to a fiberoptic spectrometer (USB 4000; Ocean Insight, Orlando, FL, USA) (Fig 1b).^24,36^ The light tip was positioned at the 12.5 mm diameter entrance to the integrating sphere, and then all of the light emitted by the LCU tip was captured. The radiant exitance (mW/cm²) was then calculated as the quotient of the average of the five radiant power values and the internal area of the LCU tip.

### Beam Profile

The radiant exitance distribution across the light tip was captured using a laser beam profiler charge-coupled device (CCD) digital camera with a 50 mm focal length lens (SP620U; Ophir-Spiricon, Logan, UT, USA) that was fixed at the focal distance from the diffusing surface of a 60° holographic diffuser target (54-505; Edmund Optics, Barrington, NJ, USA) (Fig 1c). Two blue glass bandpass filters (34-434; Edmund Optics) and one reflective neutral density filter (Edmund Optics) were required to flatten the spectral response of the CCD camera. The beam profiler camera captured the recorded image using the beam analyzer software (BeamGage Professional version 6.14, Ophir-Spiricon). The beam profile images of the LCUs could be compared qualitatively; all the images were made at the same distance using the same exposure time.

### Simulated Light-curing on MOD in Molar Teeth and the Veneer in Central Incisor

The beam profile images of the four LCUs were superposed over the image of a mesio-occluso-distal (MOD) cavity prepared on the molar tooth and over a maxillary central incisor tooth (Fig 1d).^28^

### Ergonomic Design of LCU to Access the Molar Teeth with Different Interincisal Mouth Opening Conditions

The position of the LCU over the occlusal surface of the first molar in the mannequin mouth was adjusted to 25 mm and 45 mm interincisal mouth opening conditions (Fig 1e).^25^ The LCU was fixed and stabilized with a rigid clamp device (ODEME, Luzerna, SC, Brazil). ImageJ software (developed by Wayne Rasband, National Institutes of Health, USA) measured the angle between the LCU tip and the molar’s occlusal surface (n = 5).

### Temperature Rise Measurement

**Fig 2:**
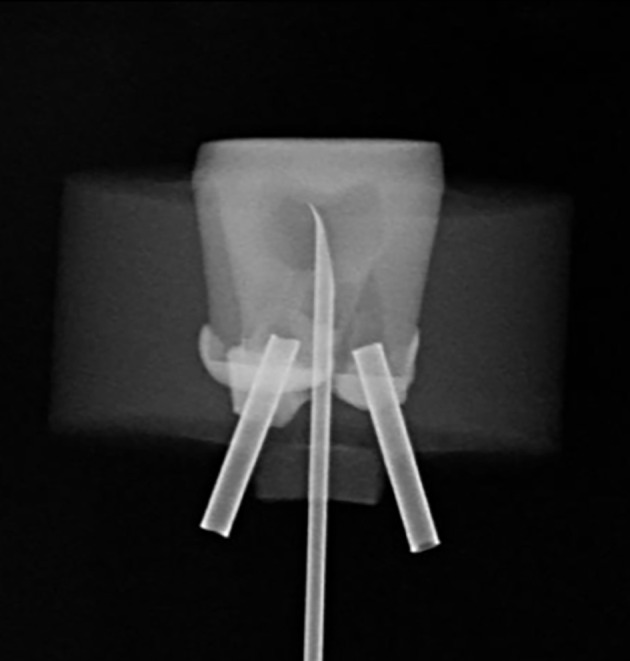
Periapical radiograph of the tooth specimen with two metal tubes and a thermocouple in contact with the top of the pulp chamber floor.

The temperature rises inside the pulp chamber of superior molar teeth with a flat cavity with 1.0 mm dentin at the pulp floor remaining (approval by the Research Ethics Committee of the Federal University of Uberlândia, CAAE approval: #49587315.4.0000.5152) during the light exposures was measured using an oral temperature simulator (Flux 100, ODEME, Luzerna, SC, Brazil).^36^ The equipment has a chamber surrounded by acrylic panels to maintain the temperature to 37°C ± 2 and 90 ± 3% relative humidity,^32,41^ thus simulating the oral environment (Fig 1f). A J-type thermocouple with a 10 Hz response time (Ecil, Piedade, SP, Brazil) was connected to the Flux 100 and was inserted into the pulp chamber through a perforation in the furcation region, maintaining contact with the pulp dentin at the top of the pulp chamber (Fig 2). This J-type thermocouple can measure temperature variations from 0 to 480°C. Real-time data were recorded at 1 Hz and exported to a computer using a dedicated software interface (ODEME). When the initial temperature had stabilized to 35°C, the experiment was started. First, the temperature of the pulp was recorded, and the light exposure was performed for 20 s in the standard mode for all LCUs and high mode of Bluephase, 5 s for the high mode for Quazar, and 3 s for the high mode of VALO Grand and VALO Cordless. The pulp temperature was recorded until the temperature returned to 37°C ± 2. The temperature peak was collected (n = 5).

### Statistical Analysis

Data were first analyzed for normal distribution (Shapiro–Wilk test) and homoscedasticity (Levene’s test). The Pearson correlation test was used to verify if there was a correlation between power, radiant exitance, and tip diameter and the market cost of the LCUs. All tests used α = 0.05 significance level, and all analyses were carried out with the statistical package Sigma Plot version 13.1 (Systat Software, San Jose, CA, USA).

## RESULTS

The external and internal diameters of the evaluated LCU tips are shown in Table 1. The external diameter ranged from 9.8 mm to 15.1 mm, with the largest value recorded for the VALO Grand and the smallest for the Bluephase G2. The internal diameter varied from 9.0 mm to 11.8 mm, with the VALO Grand having the widest light output aperture and the Bluephase G2 the narrowest. Quazar (9.9 mm) and VALO Cordless (9.7 mm) exhibited intermediate values. In Brazil, these LCUs cost US$876 to US$1515.

**Fig 3:**
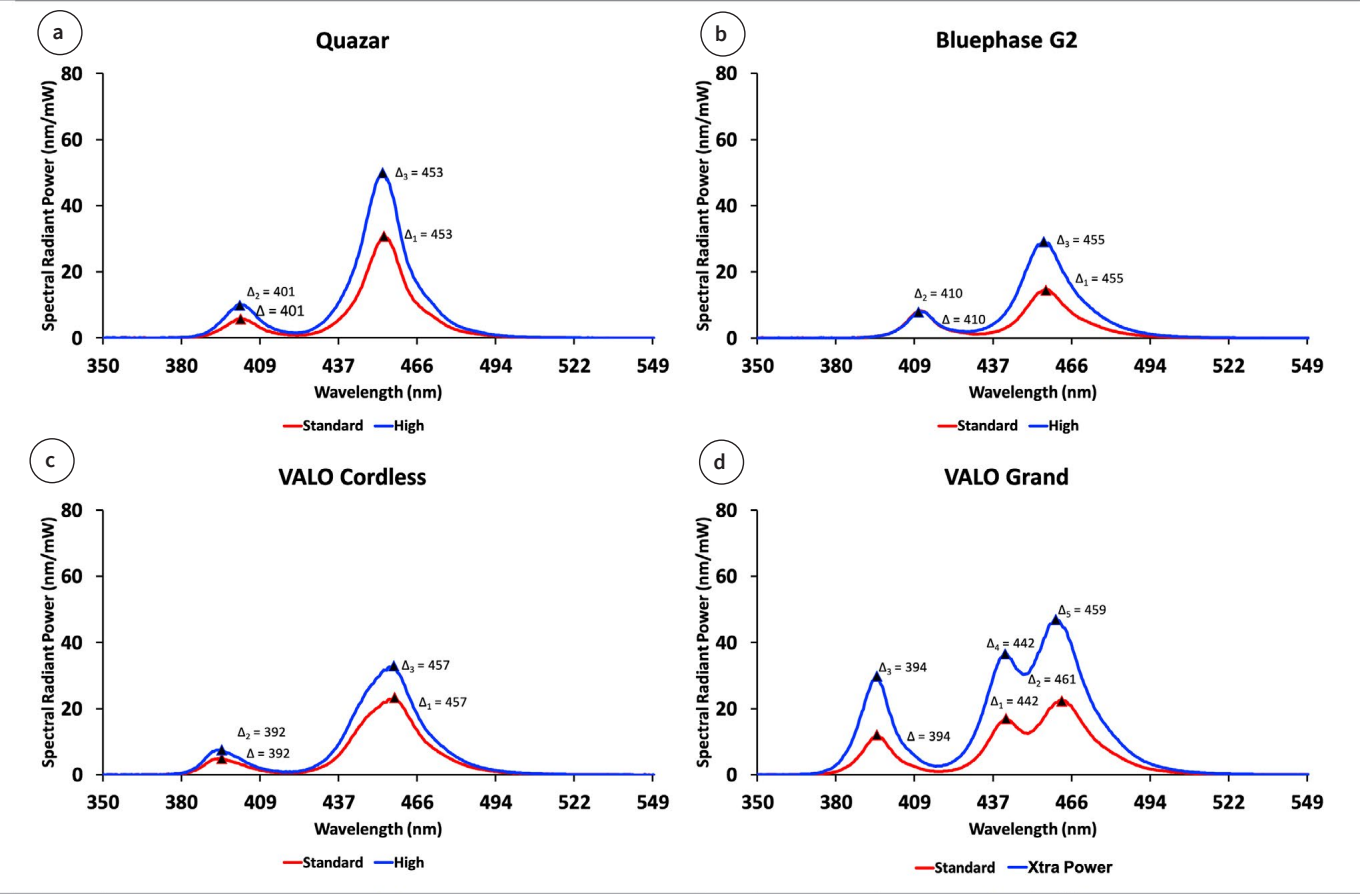
Emission spectra (nm) in the standard and high mode from the LCUs used: (a) Quazar; (b) Bluephase G2; (c) VALO Cordless; and (d) VALO Grand. The triangle indicates the absorption spectral peak of each light. All graphs were set to the same scale.

Figure 3 shows the emission spectrum of all LCUs. Quazar, Bluephase G2, and VALO Cordless delivered violet and blue light absorption peaks. VALO Grand delivered violet and blue lights, and additionally, had a third peak in the blue wavelength range. The location of these violet and blue light emission peaks was identified (Quazar: violet = 401 nm and blue = 453 nm; Bluephase G2: violet = 410 nm and blue = 455 nm; VALO Cordless: vailet – 392 nm and violet = 457 nm; VALO Grand: violet = 394 nm, blue 1 = 442 nm and blue 2 = 461 nm in the standard mode and 459 in the High or Xtra modes). The emission spectra were different between manufacturers, but they were the same between modes for each LCU used.

**Fig 4:**
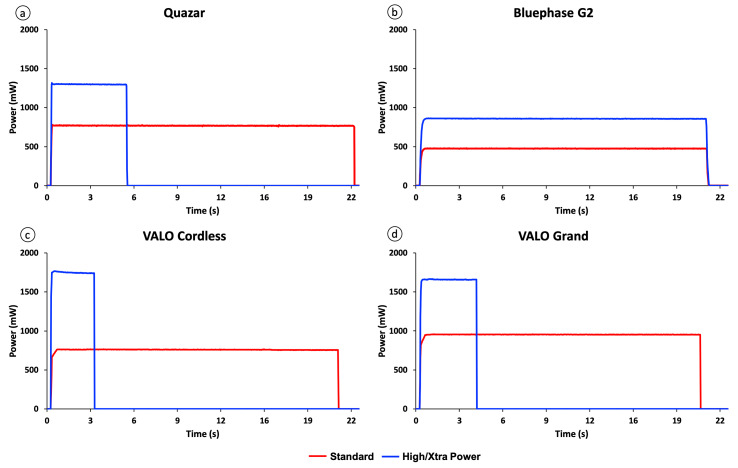
Radiant power (mW) emitted by all LCUs in standard mode for a 20-s exposure time, by the Bluephase G2 in high mode for a 20-s exposure time, by the Quazar in high mode for a 5-s exposure time, and by the VALO Cordless in high mode and Grand in Xtra power mode for a 3-s exposure time. (a) Quazar; (b) Bluephase G2; (c) VALO Cordless; and (d) VALO Grand.

Table 2 reports the power (mW), the radiant exitance (mW/cm²), and the radiant exposure (J/cm²) delivered from all the LCUs tested using standard and high/Xtra modes. Figure 4 illustrates the power (mW) for all tested LCUs. Regardless of the mode, the power values (mW) were higher for VALO Grand and lower for Bluephase G2. In standard mode, VALO Cordless and Quazar delivered higher radiant exitance (mW/cm²) and radiant exposure (J/cm²) values, while Bluephase G2 delivered the lowest values. In high/Xtra mode, VALO Cordless and Quazar delivered higher radiant exitance (mW/cm²); however, Bluephase G2 delivered higher radiant exposure (J/cm²) values due to longer exposure time.

**Table 2:** Means and standard deviation values of power (mW), radiant exitance (mW/cm²), and radiant exposure (J/cm²) of the LCUs used

LCU	Standard mode			High mode		
	Power (mW)	Radiant exitance (mW/cm²)	Radiant exposure (J/cm²)	Power (mW)	Radiant exitance (mW/cm²)	Radiant exposure (J/cm²)
Quazar	767.1 (0.9) B	996.5 (1.1) A	19.9 (0.1) A	1298.7 (1.2) B	1686.9 (1.6) A	8.4 (0.1)
Bluephase G2	469.4 (7.5) D	737.8 (11.8) C	14.8 (0.2) C	860.4 (2.6) D	1352.3 (3.3) C	27.0 (0.1)
VALO Cordless	758.1 (3.1) C	1025.7 (4.2) A	20.9 (0.1) A	1092.3 (3.1) C	1508.8 (4.4) B	4.5 (0.1)
VALO Grand	1025.8 (14.2) A	938.1 (18.4) B	18.8 (0.4) B	1656.4 (24.4) A	1514.5 (31.8) B	4.6 (0.1)
Uppercase letters compare power and radiant exitance values between the LCUs. *Radiant exposure was calculated using the specific exposure time for each.						

**Fig 5:**
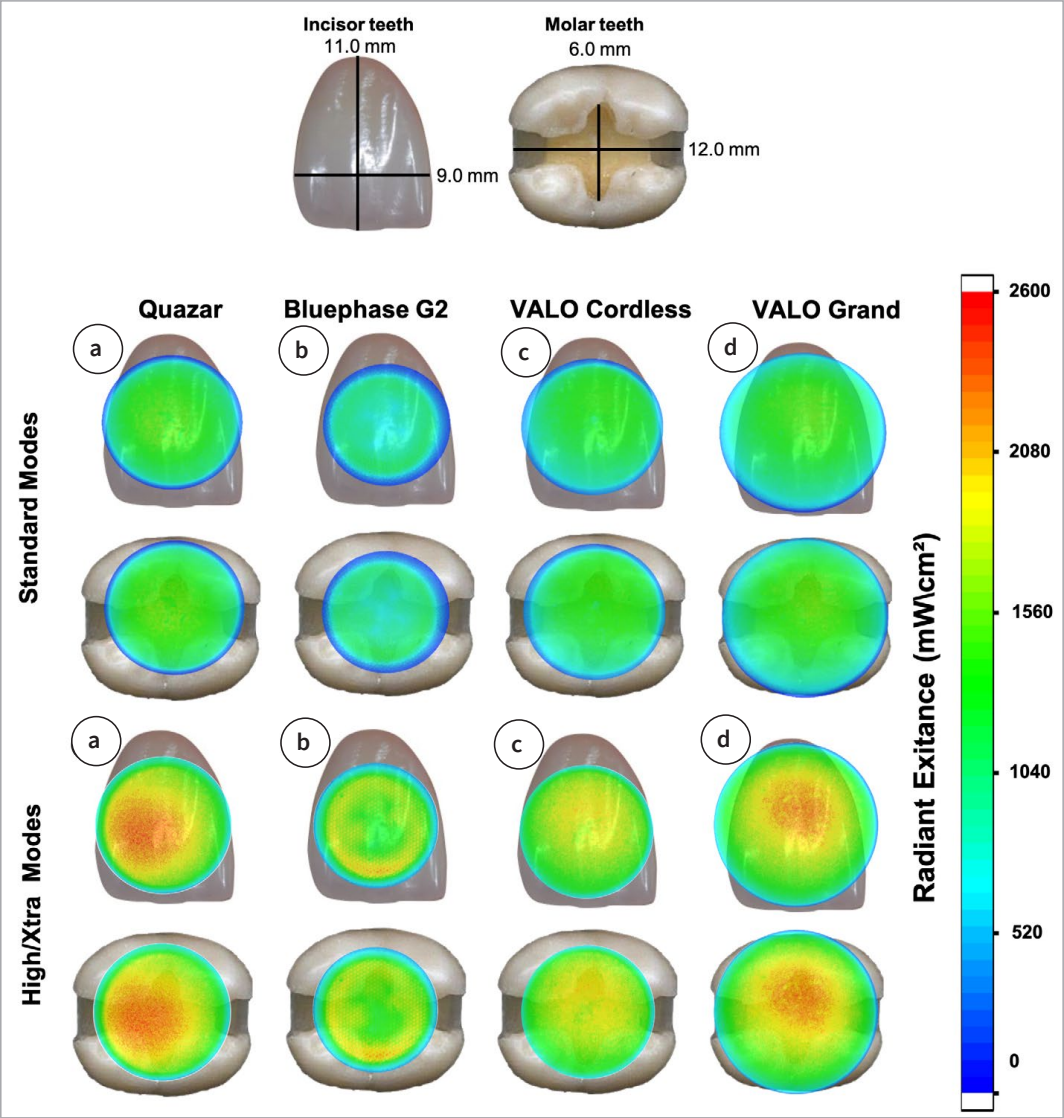
The beam profiles of calibrated radiant exitance (mW/cm²) from all LCUs in Standard and High modes are superimposed on the facial surface of a maxillary central incisor and on a MOD cavity preparation in a molar. (a) Quazar; (b) Bluephase G2; (c) VALO Cordless; and (d) VALO Grand. The images are in the same scale.

Figure 5 shows the calibrated 3D beam profiles of the LCUs, which show the tip diameter and the distribution of the average radiant output (radiant exitance in mW/cm²) across the light tip. VALO Grand covers the largest area over the maxillary incisors and molar teeth, while Bluephase covers the smallest area. In standard mode, the Quazar LCU had a hotspot area with a power of 1,600 mW/cm², while the Bluephase was homogeneous, with most types delivering a radiant exitance between 640 and 960 mW/cm². The light distribution pattern for high mode had a higher light output intensity, reaching up to 2,600 mW/cm² in some regions for Quazar, Bluephase G2 and VALO Grand. VALO Cordless showed a more homogeneous light distribution of 1,000 to 2,000 mW/cm².

**Fig 6:**
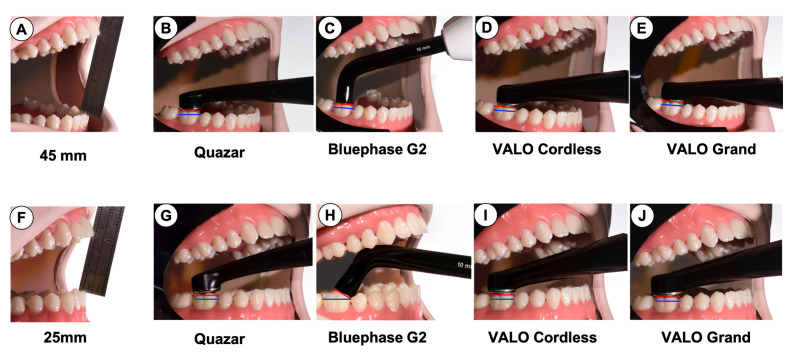
Light-curing units positioned on a first molar tooth with the patient simulator adjusted for: (a) 45 mm interincisal mouth opening with (b) Quazar; (c) Bluephase G2; (d) VALO Cordless; (e) VALO Grand positioned; and (f) 25 mm interincisal mouth opening with (g) Quazar; (h) Bluephase G2; (i) VALO Cordless, and (i) VALO Grand

Figure 6 shows the effect of different mouth openings (Fig 6a/25 mm and F/45 mm) on the positioning of the LCUs. Their angulation relative to the occlusal surface is shown in Figures 6b to e and g to j. The Quazar, VALO Grand, and VALO Cordless had a straight body design that allowed the light tip to be positioned perpendicular over the second molar tooth at both openings. At both 25 mm and 45 mm, these LCUs maintained a consistent 0-degree angle between the occlusal surface and the LCU tip, ensuring optimal light delivery. In contrast, Bluephase G2, with its angled light guide, required a notable change in tip angulation at the 25 mm interincisal mouth opening. At 25 mm, the angulation between the occlusal surface and the LCU tip increased significantly to 31.6 degrees, as indicated by the red and blue lines in Figure 6j.

**Fig 7:**
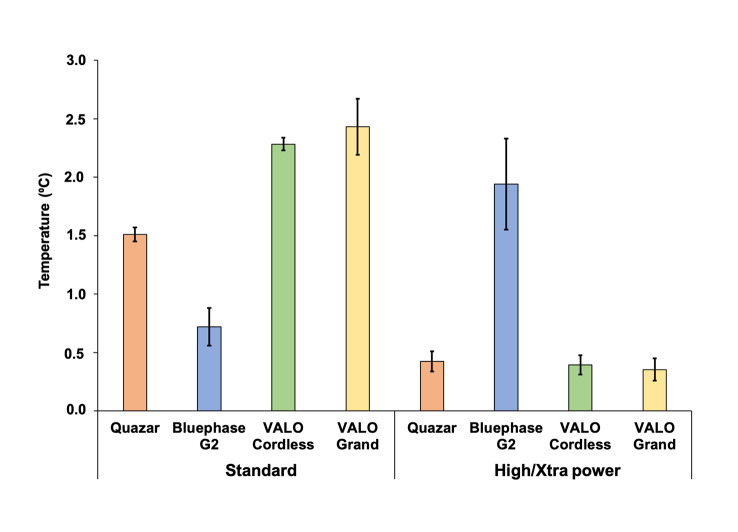
Means and standard deviations of maximum temperature rise (°C) when light-curing using the four LCUs in two different modes. For standard mode, the exposure time was 20 s for all LCUs. For high mode, the exposure time was 5 s for Quazar, 20 s for Bluephase, and 3 s for Valo Cordless and Valo Grand.

The mean temperature values and standard deviations for the different LCUs and activation modes are reported in Figure 7. In the standard mode, the VALO Grand and VALO Cordless produced the highest temperature increases, exceeding 2°C, while Bluephase G2 produced a lower temperature rise. In contrast, in the high mode, all LCUs produced significantly lower temperature increases, with values close to 0.5°C, except for Bluephase G2, which had a noticeable increase in high mode (note that the exposure time of the Bluephase in the high mode was 20 s).

The Pearson correlations among LCU cost, tip diameter, radiant power, and radiant exitance. A significant positive correlation was observed between LCU cost and internal tip diameter (P < 0.001, r = 0.81), radiant power (P < 0.001, r = 0.491), and the radiant exitance (P < 0.001, r = 0.72).

## DISCUSSION

The light-curing protocol is a critical step when restoring a tooth. Understanding the characteristics of the LCU should ensure that this step is performed to its fullest potential. The results of this study showed that the values of the tip diameter, radiant power, radiant exitance, emission spectrum, radiant exposure, and temperature rise in the pulp significantly differed between the LCUs. Some of these factors were correlated with the cost of these LCUs. The impact of design on access to the posterior region was similar between all the LCUs at the 45 mm interincisal opening. In contrast, at the 25 mm interincisal opening, the angle between the tooth and the occlusal surface increased for Bluephase G2. Therefore, the first null hypothesis was rejected.

It is essential that clinicians know the size of the active LCU tip from where light is actually emitted. The external tip diameter is not this dimension and extensive procedures, such as luting veneers in the anterior teeth or light-curing bulk-fill RBCs in mesio-occlusal-distal cavities in posterior teeth, the light tip may not cover the entire area of the restoration.^28,36^ The LCUs evaluated in this study had different tip diameter sizes: Quazar (9.9 mm), Bluephase G2 (9.0 mm), VALO Cordless (9.7 mm), and VALO Grand (11.8 mm). Even the device with the largest active tip (VALO Grand – 11.8 mm) was unable to completely cover the area of a central incisor or MOD cavity (Fig 5), requiring at least two overlapping exposures to deliver adequate light-cure.^28^ The Quazar (FGM), had a tip size comparable to that of widely established LCUs, such as the VALO Cordless, although it is still smaller than that of the VALO Grand. These findings highlight the importance of considering the diameter of the active tip when selecting an LCU to ensure that the entire surface of the tooth receives at least one light exposure.

All the LCUs used in this study have multiple emission peaks, ie, emitting light in two or more wavelengths: violet and blue. Each of the four LCUs has only one peak in the violet and blue bands, with the exception of the VALO Grand, which has two distinct peaks in the blue spectrum. The maximum wavelength peaks vary between the LCUs but remain the same between the standard and high modes of the same LCU, except for VALO Grand (Fig 3). The manufacturers of these LCUs make restorative materials that require photoactivation. FGM, for example, uses the patented advanced polymerization system (APS), which can be activated by light between 400 and 500 nm. It does appear that the Quazar LCU meets the requirements for activating FGM’s own products. Ivoclar Vivadent, the manufacturer of Bluephase LCU, uses the patented alternative photoinitiator Ivocerin as well as CQ. This photoinitiator has a maximum absorption peak at 412 nm, meaning that all LCUs tested are compatible with materials containing it, because it can be activated by wavelengths up to 460 nm. The LCUs from Ultradent have the emission ranging between 385 and 515 nm, which meets the requirements for activating all products.

The light output was stable for all the LCUs tested, regardless of the mode used (Fig 4),showing that these LCUs were able to compensate for battery power fluctuations and could maintain constant light output throughout the light exposure.^2,5,29^ The power values varied between the units, higher for VALO Grand and lower for Bluephase G2, regardless of the mode used (Table 2). The high mode differs in exposure time for the LCUs: 20 s for Bluephase, 5 s for Quazar, and 3 s for VALO Grand and VALO Cordless, as predetermined by the manufacturers, and does not correspond to the highest mode, except for Bluephase. The current trend among LCU and RBC manufacturers is to reduce exposure time. However, clinicians should understand that an intermediate power LCU that has a small tip covers the tooth with sufficient light in one exposure.^31^ Despite their high power output (mW), the LCUs tested did not deliver very high radiant exitance values (mW/cm²) due to their larger-sized tips. This contrasts with the low-cost LCUs available on the market, which often collimate the light at a particular point, resulting in high radiant exitance values over a small area, giving the false impression that they can photoactivate large restoration in one short exposure.^36^ Knowing the radiant exposure delivered to the restorative material is more important than knowing the radiant exitance at the tip of the LCU. Quazar (19.9 J/cm²), VALO Grand (20.9 J/cm²), and VALO Cordless (18.8 J/cm²) met the recommended minimum radiant exposure of 16 J/cm² required by most RBCs when used in their standard mode. However, caution should be exercised when using the high/Xtra mode, as it does not deliver the minimum energy required for 5 s for Quazar or 3 s for VALO Cordless and VALO Grand, which did not deliver the 16 J/cm². The Bluephase G2 (14.8 J/cm²) delivered lower than the minimum radiant exposure 16 J/cm² required in 20 s.

Figure 5 shows the radiant exitance distribution over the active tip area of the maxillary incisors and molars. Although the distribution was homogeneous across all LCUs, it varied based on the production strategy and activation mode. Some areas showed hotspots that reached 960 mW/cm² (Bluephase), 1,600 mW/cm² (Quazar) in standard mode, and peak values up to 2,600 mW/cm² were observed for Quazar, Bluephase G2, and VALO Grand in high mode. None of the tips could cover the entire tooth, so more than one light exposure will be required to deliver one light exposure to all of the tooth.

Increasing the distance can affect the irradiance,^7^ but the rate of decrease varies greatly with LCU design,^23^ and beam collimation. All four LCUs could be positioned directly over the molar tooth when the interincisal opening was 45 mm. However, at the 25 mm interincisal opening, the Bluephase G2 tip had to be angled (Fig 6). This may cause shadow areas at the time of photoactivation.^15,18,25^ The manufacturer of Bluephase G2 has since developed a less angled body for the Bluephase G4 and PowerCure to overcome this problem.

Raising the temperature of the pulp chamber above 5.5°C can cause irreversible damage, such as pulp necrosis and inflammatory reactions.^41^ Figure 7 shows the light-activation temperature in the two modes studied in restorations with 0.5 mm of remaining dentin. All conditions were safe as the highest temperature reached was 2.4°C in standard mode for VALO Grand. Although overlapping light-curing was suggested in this study to cover the entire area of extensive restorations, the temperature was not measured in this way. Some precautions should be taken to avoid exceeding this temperature, such as waiting for the area to cool down or directing a stream of water across the tooth.^27^

The results of this study indicate that the price (> $900) of the LCUs had a direct correlation with the radiant power values, the size of the LCU tip,^36^ suggesting that manufacturers may price devices based on their performance. Therefore, the second null hypothesis was rejected.

The study showed that there were differences in LCU performance, with variations in tip diameter, radiant power, radiant exitance, emission spectrum, and temperature rise. No single LCU tip was sufficient to cover an entire central incisor or MOD cavity in one exposure, requiring multiple overlapping activations to ensure uniform light-curing. Although temperature rise remained within safe limits, cumulative heating effects were not assessed. Lastly, the LCU price was not directly linked to radiant power or exitance. Still, it was influenced by design factors such as tip size, suggesting that clinicians should prioritize technical specifications over cost alone when selecting an LCU. The limitations of this study included that it did not examine the cumulative heating effects of multiple light-curing cycles on the increase in pulp temperature, and the effect on the pulp tissues was not examined. Also, the starting temperature was 37°C, which is not the temperature inside the pulp. Although attempts were made to standardize the remaining dentin thickness to 0.5 mm, the dentin was not uniformly 0.5 mm thick. Only four LCUs were tested, and they were tested at a 0 mm distance. Although the focus was on optical properties, the effectiveness of RBC polymerization was not evaluated. Furthermore, the beam profile analysis did not account for variations in clinical angulation, spectral inhomogeneity, or distance, indicating a need for additional investigation.

## CONCLUSION

Within the limitations of this study, it is possible to conclude that:

1. No tested LCU tip covered entirely the restoration in only one exposure.
2. Light output was stable, and all LCUs tested showed acceptable radiant exposure requirements in standard mode.
3. LCU price was linked to the internal tip diameter only.
4. Bluephase G2 created a significantly higher angulation increase at 25 mm of mouth opening than all other LCUs.
5. The Quazar LCU produced a light output that was comparable to that from leading LCUs.

### Acknowledgments

The present study was supported by the National Council for Scientific and Technological Development – CNPq grant INCT Saúde Oral e Odontologia 406840/2022-9; 140615/2021-0 and 422603/2021-0; and by the Research Support Foundation of the State of Minas Gerais – FAPEMIG grant number APQ-02105-18 Rede Mineira de Saúde Oral e Odontologia grant FAPEMIG number RED-00204-23, and FAPEMIG/DPT nº.57386799/2022. The authors extend their thanks to CPBio and the BIAOR Research Group for their valuable contributions to this work.

#### Clinical relevance

No LCU tip fully covers a restoration in a single exposure. The price is linked to design rather than performance. Selecting an LCU should prioritize efficiency, ergonomic factors, and light distribution rather than cost.

## References

[ref1] AlShaafi MM, AlQussier A, AlQahtani MQ, Price RB (2018). Effect of mold type and diameter on the depth of cure of three resin-based composites. Oper Dent.

[ref2] AlShaafi MM, Harlow JE, Price HL (2016). Emission characteristics and effect of battery drain in “budget” curing lights. Oper Dent.

[ref3] Barcelos LM, Braga S, Pereira R, Price RB, Soares CJ (2023). Effect of using manufacturer-recommended exposure times to photo-activate bulk-fill and conventional resin-based composites. Oper Dent.

[ref4] Braga S, Oliveira L, Ribeiro M (2019). Effect of simulated pulpal microcirculation on temperature when light curing bulk fill composites. Oper Dent.

[ref5] Cardoso IO, Machado AC, Teixeira D, Basilio FC, Marletta A, Soares PV (2020). Influence of different cordless light-emitting-diode units and battery levels on chemical, mechanical, and physical properties of composite resin. Oper Dent.

[ref6] Contreras SCM, Jurema ALB, Claudino ES, Bresciani E, Caneppele TMF (2021). Monowave and polywave light-curing of bulk-fill resin composites: degree of conversion and marginal adaptation following thermomechanical aging. Biomater Investig Dent.

[ref7] de Deus RA, Oliveira L, Braga S, Ribeiro M, Price RB, Nunez A (2024). Effect of radiant exposure on the physical and mechanical properties of 10 flowable and high-viscosity bulk-fill resin composites. Oper Dent.

[ref8] de Oliveira D, Rocha MG, Correa IC, Correr AB, Ferracane JL, Sinhoreti MAC (2016). The effect of combining photoinitiator systems on the color and curing profile of resin-based composites. Dent Mater.

[ref9] Ferracane JL (2024). A historical perspective on dental composite restorative materials. J Funct Biomater.

[ref10] Guarneri JAG, Price RB, Maucoski C, Arrais CAG (2024). The dark art of light curing in dentistry. J Dent.

[ref11] Hadis MA, Shortall AC, Palin WM (2024). The power of light – from dental materials processing to diagnostics and therapeutics. Biomater Investig Dent.

[ref12] Harlow JE, Rueggeberg FA, Labrie D, Sullivan B, Price RB (2016). Transmission of violet and blue light through conventional (layered) and bulk cured resin-based composites. J Dent.

[ref13] Hasanain FA, Nassar HM (2021). Utilizing light cure units: a concise narrative review. Polymers (Basel).

[ref15] Konerding KL, Heyder M, Kranz S, Guellmar A, Voelpel A, Watts DC (2016). Study of energy transfer by different light curing units into a class III restoration as a function of tilt angle and distance, using a MARC Patient Simulator (PS). Dent Mater.

[ref16] Kowalska A, Sokolowski J, Bociong K (2021). The photoinitiators used in resin based dental composite – a review and future perspectives. Polymers (Basel).

[ref17] Kowalska-Kuczynska A, Sokolowski J, Szynkowska-Jozwik MI (2023). Evaluation of the selected mechanical and aesthetic properties of experimental resin dental composites containing 1-phenyl-1,2-propanedione or phenylbis(2,4,6-trimethylbenzoyl)-phosphine oxide as a photoinitiator. Int J Mol Sci.

[ref18] Langford DK, Wells MH, Vinall CV, Tantbirojn D, Versluis A (2024). Using bulk-fill composite and high-intensity curing when light tip placement is compromised. Pediatr Dent.

[ref19] Lara L, Rocha MG, Menezes LR, Correr AB, Sinhoreti MAC, Oliveira D (2021). Effect of combining photoinitiators on cure efficiency of dental resin-based composites. J Appl Oral Sci.

[ref20] Lim HK, Keerthana S, Song SY, Li C, Shim JS, Ryu JJ (2024). Effect of light irradiance and curing duration on degree of conversion of dual-cure resin core in various cavities with different depths and diameters. Materials (Basel).

[ref21] Maktabi H, Ibrahim MS, Balhaddad AA, Alkhubaizi Q, Garcia IM, Collares FM (2021). Improper light curing of bulkfill composite drives surface changes and increases S. mutans biofilm growth as a pathway for higher risk of recurrent caries around restorations. Dent J (Basel).

[ref22] Malhotra S, Kaur R, Saroa PK, Kaur K, Sandhu KK, Thukral V (2023). Effect of curing distance for cure depth in composite resin. Bioinformation.

[ref23] Maucoski C, Price RB, Arrais CA, Sullivan B (2022). Power output from 12 brands of contemporary LED light-curing units measured using 2 brands of radiometers. PLoS One.

[ref24] Michaud PL, Price RB, Labrie D, Rueggeberg FA, Sullivan B (2014). Localised irradiance distribution found in dental light curing units. J Dent.

[ref25] Moreira RJ, de Deus RA, Ribeiro MTH, Braga SSL, Schettini ACT (2021). Effect of light-curing unit design and mouth opening on the polymerization of bulk-fill resin-based composite restorations in molars. J Adhes Dent.

[ref26] Oh S, Kim HJ, Kim HJ, Antonson SA, Kim SY (2022). Influence of irradiation distance on the mechanical performances of resin composites polymerized with high-irradiance light curing units. Biomater Res.

[ref27] Onisor I, Asmussen E, Krejci I (2011). Temperature rise during photo-polymerization for onlay luting. Am J Dent.

[ref28] Peres TS, de Quirino Oliveira HL, Mendoza LCL (2024). Effect of four different mono and multi-wave light-curing units on the Knoop hardness of veneer resin composites. Dent Mater.

[ref29] Peres TS, Oliveira G, da Silva Sakamoto SP, da Silva Faria M, Carlo HL, Soares CJ (2024). Effect of battery level during successive charging cycles on the performance of certified and low-cost uncertified light-curing units available on E-commerce. Oper Dent.

[ref30] Pratap B, Gupta RK, Bhardwaj B, Nag M (2019). Resin based restorative dental materials: characteristics and future perspectives. Jpn Dent Sci Rev.

[ref31] Ribeiro M, Maucoski C, Price RB, Soares CJ (2024). Effect of a 3-second off-label exposure on the depth of cure of eight resin-based composites. Oper Dent.

[ref32] Runnacles P, Arrais CA, Pochapski MT, Dos Santos FA, Coelho U, Gomes JC (2015). In vivo temperature rise in anesthetized human pulp during exposure to a polywave LED light curing unit. Dent Mater.

[ref34] Shimokawa C, Sullivan B, Turbino ML, Soares CJ, Price RB (2017). Influence of emission spectrum and irradiance on light curing of resin-based composites. Oper Dent.

[ref35] Shortall AC, Hadis MA, Palin WM (2021). On the inaccuracies of dental radiometers. PLoS One.

[ref36] Soares CJ, Braga S, Price RB (2021). Relationship between the cost of 12 light-curing units and their radiant power, emission spectrum, radiant exitance, and beam profile. Oper Dent.

[ref37] Soares CJ, Braganca GF, Pereira R, Rodrigues MP, Braga SSL, Oliveira LRS (2018). Irradiance and radiant exposures delivered by LED light-curing units used by a left and right-handed operator. Braz Dent J.

[ref38] Soares CJ, Rodrigues MP, Oliveira LRS, Braga SSL, Barcelos LM, Silva GRD (2017). An evaluation of the light output from 22 contemporary light curing units. Braz Dent J.

[ref39] Thyvalikakath T, Siddiqui ZA, Eckert G, LaPradd M, Duncan WD, Gordan VV (2024). Survival analysis of posterior composite restorations in National Dental PBRN general dentistry practices. J Dent.

[ref40] Wang WJ, Grymak A, Waddell JN, Choi JJE (2021). The effect of light curing intensity on bulk-fill composite resins: heat generation and chemomechanical properties. Biomater Investig Dent.

[ref41] Zarpellon DC, Runnacles P, Maucoski C, Gross DJ, Coelho U, Rueggeberg FA, Arrais C (2021). In vivo pulp temperature changes during class V cavity preparation and resin composite restoration in premolars. Oper Dent.

